# Explorative study for the rapid detection of Fritillaria using gas chromatography-ion mobility spectrometry

**DOI:** 10.3389/fnut.2024.1361668

**Published:** 2024-02-06

**Authors:** Yuping Dai, Shanshuo Liu, Li Yang, Ye He, Xiao Guo, Yang Ma, Shunxiang Li, Dan Huang

**Affiliations:** ^1^State Key Laboratory of Chinese Medicine Powder and Medicine Innovation in Hunan (Incubation), Science and Technology Innovation Center, Hunan University of Chinese Medicine, Changsha, China; ^2^Hunan Fenghuang Lanke Traditional Chinese Medicine Co., Ltd., Changsha, China; ^3^Hunan Engineering Technology Research Center for Bioactive Substance Dis-covery of Chinese Medicine, School of Pharmacy, Hunan University of Chinese Medicine, Changsha, China; ^4^Chongqing Healn Drug Sales Co., Ltd., Chongqing, China; ^5^Hunan Province Sino-US International Joint Research Center for Therapeutic Drugs of Senile Degenerative Diseases, Changsha, China

**Keywords:** Fritillaria, gas chromatography-ion mobility spectrometry, PCA, CA, PLS-DA

## Abstract

Fritillaria is a well-known health-promoting food, but it has many varieties and its market circulation is chaotic. In order to explore the differences in volatile organic compounds (VOCs) among different varieties of Fritillaria and quickly and accurately determine the variety of Fritillaria, this study selected six varieties of Fritillaria and identified and analyzed their volatile components using gas chromatography-ion mobility spectrometry (GC-IMS), establishing the characteristic fingerprints of VOCs in Fritillaria. In all samples, a total of 76 peaks were detected and 67 VOCs were identified. It was found that the composition of VOCs in different varieties of Fritillaria was similar, but the content was different. Combined with chemometric analysis, the differences between VOCs were clearly shown after principal component analysis, cluster analysis, and partial least-squares discriminant analysis. This may provide theoretical guidance for the identification and authenticity determination of different varieties of Fritillaria.

## 1 Introduction

Fritillaria is a well-known health-promoting food. Popular Fritillaria -based foods including Fritillaria chicken soup, Fritillaria pear paste and Fritillaria wine, have been gradually developed in the world. It belongs to the Liliaceae family, and there are about 130 species around the world; it is widely distributed in temperate regions of the northern hemisphere, especially in Central Asia, North America, and the Mediterranean region. There are about 40 species of Fritillaria in China, mainly distributed in Sichuan, Zhejiang, Gansu, Xinjiang, and Hubei and other places. It was recorded in the classis Chinese medicine work “*Shen nong’s Herbal Classis”* more than 2,000 years ago. Fritillaria tastes hot and flat, and is mainly used to treat cold and fever, drenching, hernia paralysis, laryngeal paralysis, lactation, and wind spasms. Modern research shows that it has antitumor (1, 2), antioxidant (3, 4), and antibacterial effects (5), and prevents neuropathies such as Alzheimer’s disease (6).

The current 2020 edition of the Chinese Pharmacopoeia contains eight herbs of the genus Fritillaria, which are categorized as Fritillariae cirrhosae bulbus, Fritillariae thunbergh bulbus,Fritillariae ussuriensis bulbus,Fritillariae pallidiflorae bulbus,and Fritillariae hupehensis bulbus (7). General Fritillariae cirrhosae bulbus is bittersweet and slightly cold, mostly used for deficiency coughs (8–12); Fritillariae thunbergh bulbus is bitter cold, mostly used for external coughs (13), which, in the clinical sense, is labeled “Chuanbeimu” and “Zhebeimu” according to the corresponding evidence; and Fritillariae ussuriensis bulbus (14) and Fritillariae pallidiflorae bulbus have the same efficacy as Fritillariae cirrhosae bulbus (15). Fritillariae hupehensis bulbus is used for coughs due to heat-phlegm, and for subcutaneous nodule scrofula. It can be seen that different varieties of fritillaria directly affect its quality, thus affecting its clinical efficacy (16).

Due to the special growth environment of Fritillariae cirrhosae bulbus, its growth is slow, and existing wild resources have been overharvested; however, there has been no breakthrough in artificial planting technology, resulting in the scarcity of genuine Fritillariae cirrhosae bulbus which cannot meet the market demand, prompting prices to soar. In addition, the market contains a wide range of Fritillaria medicinal materials with complex sources and similar appearances. In some cases, the cheaper species of Fritillaria (such as Fritillariae thunbergh bulbus,Fritillariae ussuriensis bulbus,Fritillariae pallidiflorae bulbus,Fritillariae hupehensis bulbus, and so on) are adulterated or sold as Fritillariae cirrhosae bulbus in order to obtain higher profits. This seriously affects the market order, jeopardizes the interests of consumers, and affects the safety and efficacy of medication. Therefore, it is very meaningful to establish an objective, scientific, accurate, and rapid method to identify different species of Fritillaria and adulteration problems (17).

At present, research into Fritillaria identification mostly uses feature and microscopic identification, DNA barcoding technique, high-performance liquid chromatography fingerprinting, etc. feature identification and microscopic identification is fast, but requires skill, and it is subjective, while DNA barcoding technique identification and high-performance liquid chromatography fingerprinting is precise and accurate, but the process is cumbersome and demanding on operators, takes a lot of time, and is expensive (18, 19).

Gas chromatography-ion mobility spectrometry (GC-IMS) is a fast and highly efficient analytical instrument that can operate under ambient pressure and temperature. It combines the strong separation capability of GC with the advantages of the high sensitivity and high resolution of IMS, which provides richer chemical information and more comprehensive analysis of compounds, as well as fast analysis speed with less sample dosage, and provides convenient conditions for the detection of samples with only trace amounts. Highly efficient and convenient conditions are provided for the detection of samples that can only be obtained in minute quantities (20). GC-IMS is now used in the fields of foods, odor analysis and environmental testing (21–24). In addition, GC-IMS technology is used to provide certain theoretical references for the identification, quality evaluation and production of medicinal herbs (25–28). Different varieties have certain differences in the types and contents of their compounds. Fritillaria contains a variety of VOCs, given that GC-IMS technology has the ability to efficiently analyze volatile substances, we predict that the type of Fritillaria can be quickly identified by analyzing the distribution and relative content of various VOCs in caladium by GC-IMS. In addition, identification accuracy is further improved by effectively identifying and quantifying various compounds. However, few studies have been reported on the identification of different species of Fritillaria using GC-IMS with Chemometric. Therefore, it is necessary to systematically and comprehensively analyze VOCs in different types Fritillaria under new technologies.

In this study, VOCs in different species of Fritillaria were studied with GC-IMS. Principal component analysis (PCA), cluster analysis (CA), and Partial least-squares discriminant analysis (PLS-DA) were used to analyze the differences of VOC odor fingerprints in different varieties of Fritillaria. The characteristic VOCs in different varieties of Fritillaria were displayed in a visual form, thus laying a certain foundation for rapid identification of Fritillaria in different species. This will be helpful to ensure the quality of Fritillaria and ensure the safety and effectiveness of medication for patients.

## 2 Materials and methods

### 2.1 Materials

The powders of the six kinds of Fritillaria were purchased from the National Institutes for Food and Drug Control, Beijing, China: Tendrilleaf Fritillary Bulb (the dried bulb of Fritillaria cirrhosa D. Don, No. 121757-202101, named CBM-01; the dried bulb of Fritillaria unibracteata Hsiao et K. C. Hsia, No. 121000-201609, named CBM-02); Thunberg Fritillary Bulb (the dried bulb of Fritillaria thunbergii Miq, No. 120972-201906, named ZBM-02); Ussuri Fritillary Bulb (the dried bulb of Fritillaria ussuriensis Maxim., No. 120924-201711, named PBM-01); Sinkiang Fritillary Bulb (the dried bulb of Fritillaria walujewii Regel, No. 121739-201701, named YBM-01); and Hubei Fritillary Bulb (the dried bulb of Fritillaria hupehensis Hsiao et K. C. Hsia, No. 120962-201005, named HBBM-01).

### 2.2 GC-IMS methods

#### 2.2.1 Sample preparation

The powders of the samples were accurately weighed out to 1 g and placed into a 20 mL headspace vial, then incubated at 80°C for 15 min.

#### 2.2.2 Headspace conditional

Static headspace autosampler unit (CTC-PAL 3), (CTC Analytics AG, Zwingen, Switzerland); injection volume: 500 μL; non-shunt injection; speed: rotation speed of 500 rpm for 20 min; injection needle temperature: 85°C.

#### 2.2.3 GC conditional

A chromatographic column of MXT-WAX (15 m × 0.53 mm, 1.0 μm, Restek Inc., United States) was used. Column temperature: 60°C; carrier gas: high-purity N_2_ (purity ≥99.999%); programmed boosting: initial flow rate of 2.00 mL/min was held for 2 min, linearly increased to 10.00 mL/min within 8 min, linearly increased to 100.00 mL/min within 10 min, and held for 10 min. Chromatographic runtime: 30 min; injection temperature: 80°C. Run time: 30 min; inlet temperature: 80°C.

#### 2.2.4 IMS conditional

GC-IMS instrument: FlavourSpec^®^ Gas Phase Ion Mobility Spectrometer, G.A.S. (Dortmund, Germany); ionization source: tritium source (3H); drift tube length: 53 mm; electric field strength: 500 V/cm; drift tube temperature: 45°C; drift gas: high-purity N_2_ (purity ≥99.999%); flow rate: 150 mL/min; positive ion mode.

### 2.3 Statistical analysis

Preliminarily, the peaks of the samples were chosen and compared using the Laboratory Analytical Viewer (v.2.2.1, G.A.S.) and Reporter analysis (v.1.2.12, G.A.S.). We performed quantitative analysis using Gallery Plot Analysis (v.1.0.7, G.A.S.) on selected signal peaks. The principal component analysis (PCA) was performed in Origin software (Northampton, Massachusetts, United States), TBtools was used for cluster analysis and partial least-squares discriminant analysis (PLS-DA) of volatile profiles was performed in SIMCA software (Umea, Sweden).

To compare VOCs among samples, Reporter, Gallery Plot, and other plug-ins in VOCal data processing software were used to generate three-dimensional spectra, two-dimensional spectra, difference spectra, and fingerprints.

## 3 Results and discussion

### 3.1 Qualitative analysis of Fritillaria samples from different species

**Figure 1:**
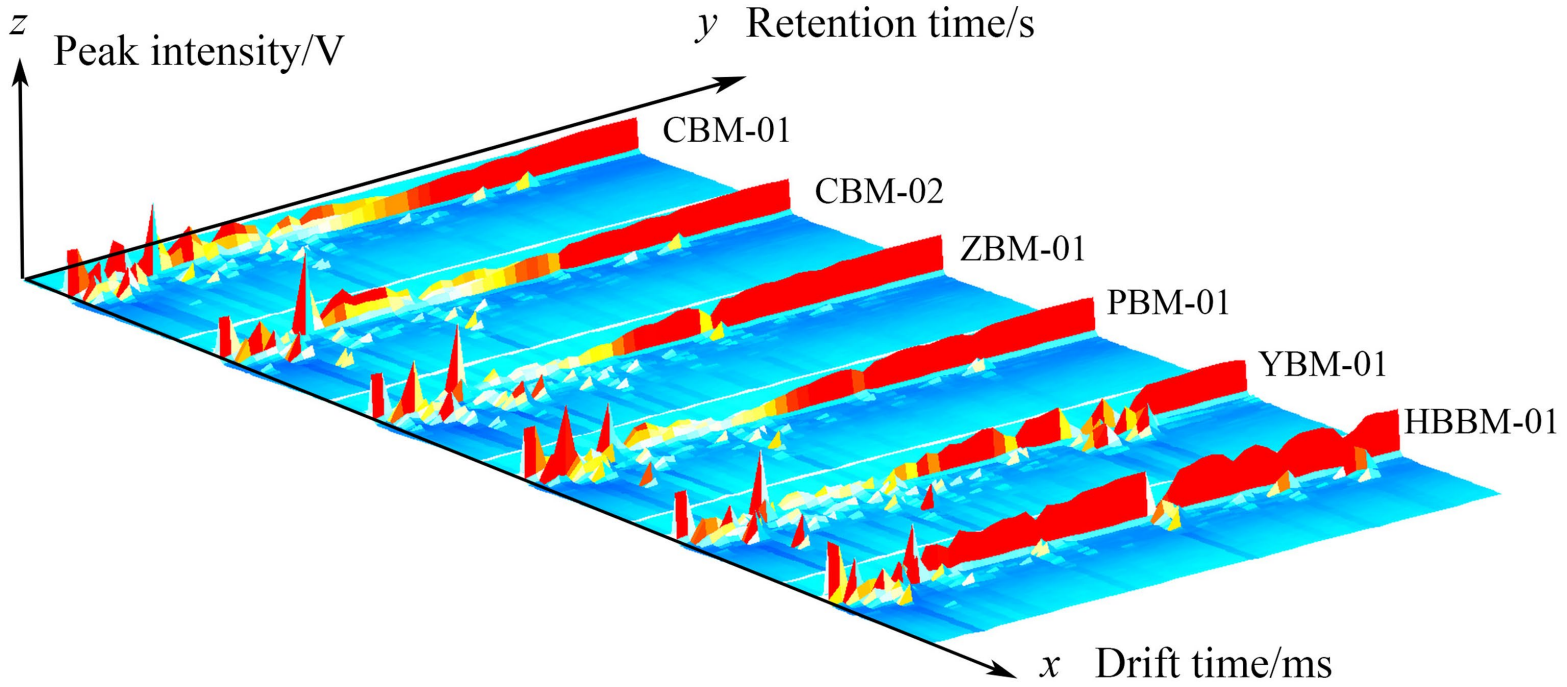
GC-IMS three-dimensional spectra of different varieties of Fritillaria.

**Figure 2:**
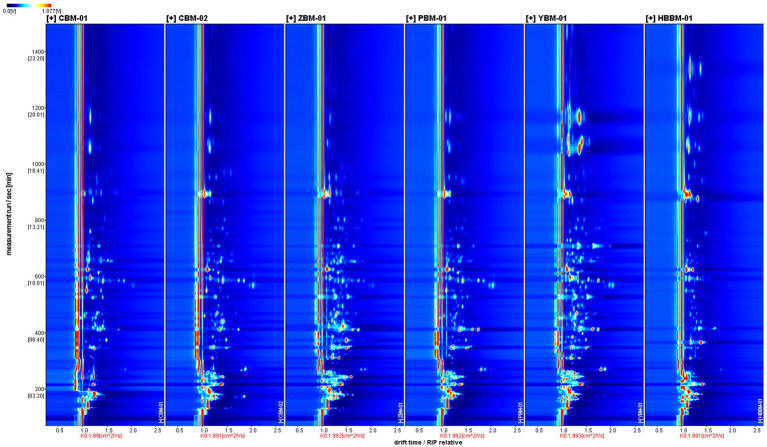
GC-IMS two-dimensional spectra of different varieties of Fritillaria.

**Figure 3:**
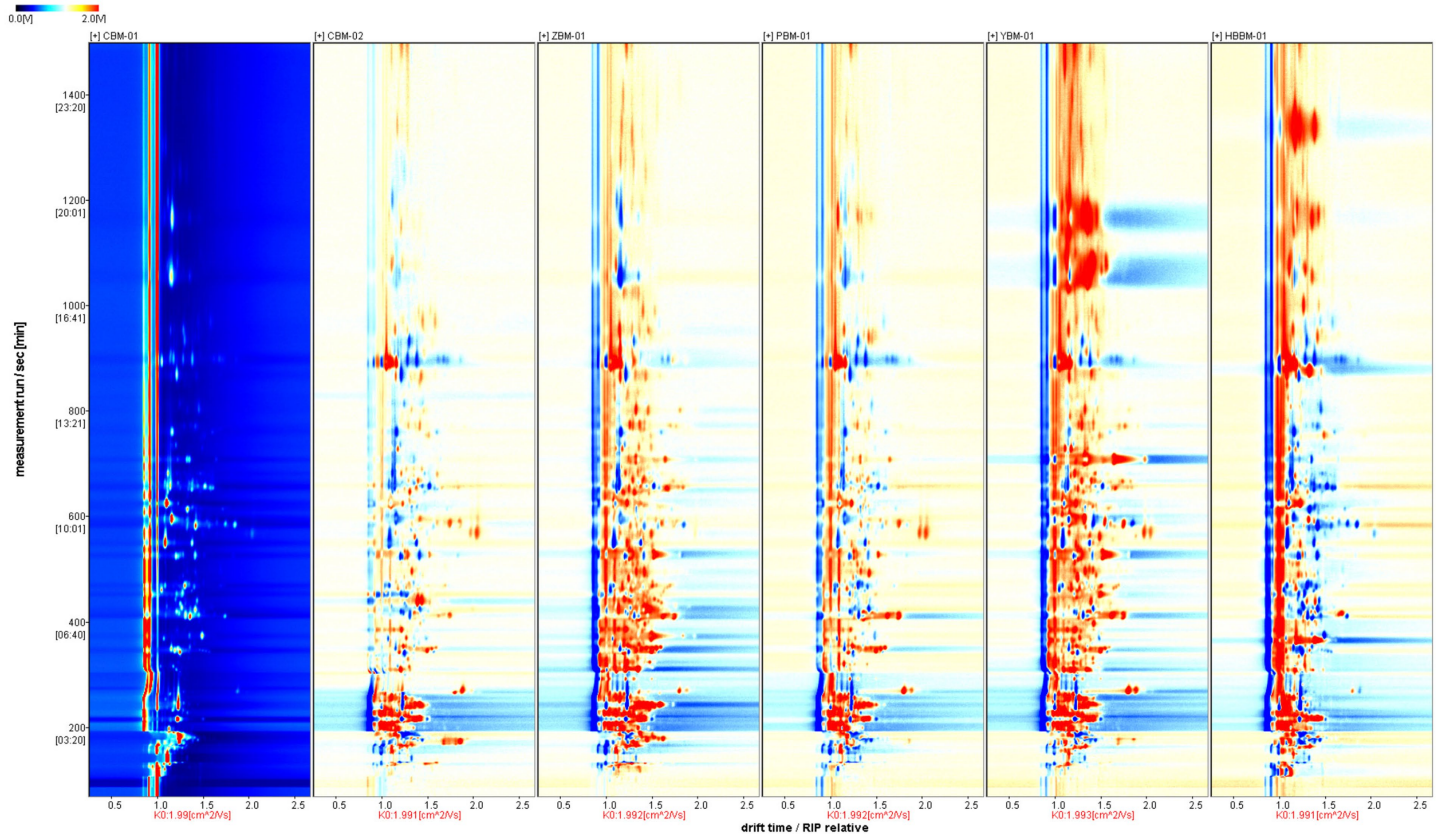
GC-IMS difference comparison chart of different varieties of Fritillaria.

The three-dimensional(3D) GC-IMS spectra of different varieties of Fritillaria are shown in Figure 1. The three coordinates represent the drift time, retention time, and signal peak intensity, respectively, from which we can visualize the differences in VOCs of different varieties of Fritillaria. Figure 2 shows a comparison of the differences: the background of the whole image is blue, the red vertical line at 1.0 of the horizontal coordinates represents the RIP (reactive ion peak: represents the total amount of available ions formed), each point on both sides of the RIP represents one VOC, and the color represents the peak intensity of the substance. From blue to red, the darker the color, the greater the peak intensity, and it can be seen that there are certain differences in the VOCs from the samples of different types of Fritillaria. In order to further visualize and compare the differences in volatile components, the spectrum of the CBM-1 sample was selected as a reference, and the spectra of other samples were deducted from the reference to obtain a comparison of the differences between different samples, as shown in Figure 3. If the VOC contents in the target sample and the reference were the same, the background after deduction was white, while a red color means that the concentration of the substance was higher than the reference in the target sample, and a blue color means that the concentration of the substance was lower than the reference in the target sample.

#### 3.1.1 Comparison of differences in volatile components of CBM-01 and CBM-02 samples

Using the comparison chart of the difference in VOCs of the CBM-01 and CBM-02 samples, it can be seen that there was also a significant difference in the VOCs. Although they are all named Tendrilleaf Fritillary Bulb, different origins, cultivation methods, and storage times may affect the content of some volatile substances.

#### 3.1.2 Comparison of the differences in VOCs between Tendrilleaf Fritillary Bulb and other varieties of Fritillaria

The VOCs in all varieties of Fritillaria were analyzed using GC-IMS and well separated. The composition of the VOCs in different varieties of Fritillaria was similar, but there were significant differences in the contents. This may also be related to the fact that different varieties of Fritillaria can exert different medicinal effects.

### 3.2 Identification of VOCs in different Fritillaria samples

**Figure 4:**
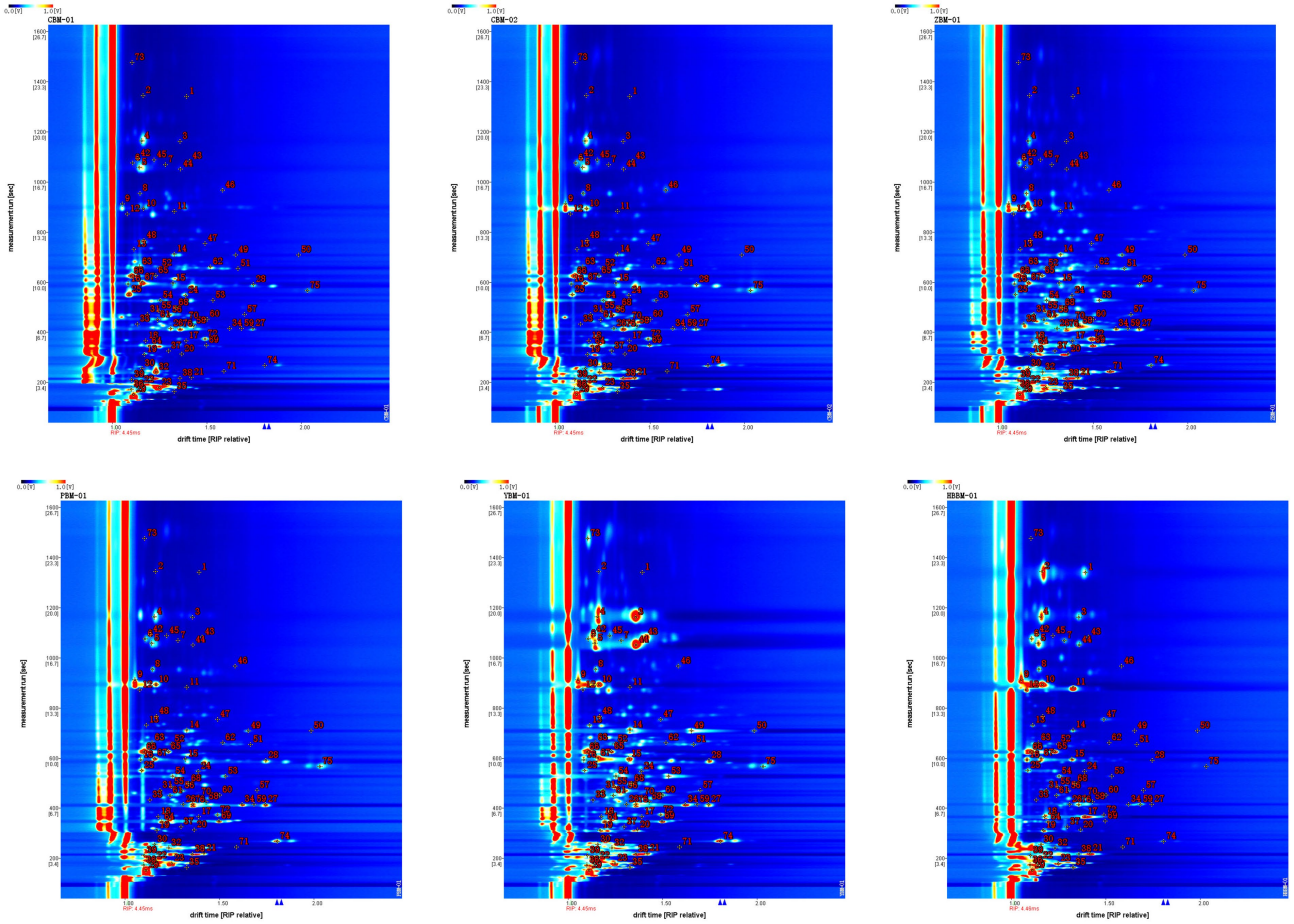
Characteristic peak position plot of volatile components of six species of Fritillaria.

After GC-IMS analysis, a total of 76 substances were detected, and two-dimensional characterization was carried out using the NIST2020 gas-phase retention index database built in the Practical Vocal software and the IMS drift time database of G.A.S. The results of the GC-IMS analysis are shown in Table 1 and Figure 4.

**Table 1:** Results of component analysis of different varieties of Fritillaria.

Count	Compound	CAS	Molecular formula	RI	Rt (sec)	Dt (a.u.)	Comment
1	1-Butanoic acid D	C107926	C_4_H_8_O_2_	1,649	1340.216	1.3959	Dimers
2	1-Butanoic acid M	C107926	C_4_H_8_O_2_	1650.1	1343.799	1.16474	Monomers
3	2-Methyl propanoic acid D	C79312	C_4_H_8_O_2_	1587.4	1161.106	1.36183	Dimers
4	2-Methyl propanoic acid M	C79312	C_4_H_8_O_2_	1588.1	1162.897	1.15744	Monomers
5	Methyl 2-furoate	C611132	C_6_H_6_O_3_	1547.2	1057.221	1.14527	–
6	Propanoic acid M	C79094	C_3_H_6_O_2_	1554.1	1074.349	1.10932	Monomers
7	Propanoic acid D	C79094	C_3_H_6_O_2_	1551.8	1068.667	1.28382	Dimers
8	Benzaldehyde	C100527	C_7_H_6_O	1502.7	953.128	1.14905	–
9	Acetic acid M	C64197	C_2_H_4_O_2_	1483.5	911.458	1.05683	Monomers
10	Acetic acid D	C64197	C_2_H_4_O_2_	1474.5	892.517	1.16891	Dimers
11	2-Furaldehyde D	C98011	C_5_H_4_O_2_	1,469	881.152	1.33063	Dimers
12	2-Furaldehyde M	C98011	C_5_H_4_O_2_	1464.4	871.682	1.08095	Monomers
13	3-Ethylpyridine	C536787	C_7_H_9_N	1388.6	730.572	1.11642	–
14	1-Hexanol M	C111273	C_6_H_14_O	1377.4	711.631	1.33063	Monomers
15	2-Butanone, 3-hydroxy D	C513860	C_4_H_8_O_2_	1300.6	595.075	1.32292	Dimers
16	2-Butanone, 3-hydroxy M	C513860	C_4_H_8_O_2_	1298.4	592.065	1.08668	Monomers
17	1-Butanol D	C71363	C_4_H_10_O	1156.1	365.866	1.39434	Dimers
18	1-Butanol M	C71363	C_4_H_10_O	1156.4	366.296	1.18007	Monomers
19	Isobutanol M	C78831	C_4_H_10_O	1109.6	311.744	1.17231	Monomers
20	Isobutanol D	C78831	C_4_H_10_O	1110.3	312.534	1.36949	Dimers
21	n-Pentanal	C110623	C_5_H_10_O	994	219.538	1.42151	–
22	Ethyl propanoate	C105373	C_5_H_10_O_2_	944.9	190.384	1.1563	–
23	2-Butanone	C78933	C_4_H_8_O	925	179.682	1.25147	–
24	2-Methylpyrazine D	C109080	C_5_H_6_N_2_	1274.1	547.149	1.39146	Dimers
25	2-Methylpyrazine M	C109080	C_5_H_6_N_2_	1,276	550.571	1.09067	Monomers
26	Amyl acetate M	C628637	C_7_H_14_O_2_	1,193	415.396	1.31099	Monomers
27	Amyl acetate D	C628637	C_7_H_14_O_2_	1192.3	414.54	1.74781	Dimers
28	2-Octanone	C111137	C_8_H_16_O	1297.1	590.208	1.75051	–
29	Acetone	C67641	C_3_H_6_O	864.8	150.836	1.11503	–
30	2-Butanol	C78922	C_4_H_10_O	1044.8	255.417	1.16148	–
31	2-Hexenal	C505577	C_6_H_10_O	1229.7	470.587	1.18911	–
32	Butyl acetate	C123864	C_6_H_12_O_2_	1024.3	240.282	1.23439	–
33	(Z)-4-Heptenal M	C6728310	C_7_H_12_O	1204.8	432.369	1.13614	Monomers
34	(Z)-4-Heptenal D	C6728310	C_7_H_12_O	1192.9	415.279	1.62404	Dimers
35	Ethyl acetate D	C141786	C_4_H_8_O_2_	893.7	164.095	1.3305	Dimers
36	Ethyl acetate M	C141786	C_4_H_8_O_2_	911.2	172.623	1.10347	Monomers
37	Isopentyl acetate	C123922	C_7_H_14_O_2_	1121.9	325.264	1.30052	–
38	3-Pentanone D	C96220	C_5_H_10_O	989.7	216.833	1.36179	Dimers
39	3-Pentanone M	C96220	C_5_H_10_O	980	210.783	1.10587	Monomers
40	γ-pentalactone M	C108292	C_5_H_8_O_2_	1561.1	1091.954	1.13706	Monomers
41	γ-pentalactone D	C108292	C_5_H_8_O_2_	1,557	1081.744	1.41232	Dimers
42	2,3-Butandiol	C513859	C_4_H_10_O_2_	1544.3	1050.075	1.3642	–
43	Linalool	C78706	C_10_H_18_O	1559.6	1088.162	1.22272	–
44	1-Octen-3-ol	C3391864	C_8_H_16_O	1508.7	966.427	1.58577	–
45	(E)-2-Hexen-1-ol	C928950	C_6_H_12_O	1402.3	754.262	1.49336	–
46	2-Ethyl-3-methylpyrazine	C15707230	C_7_H_10_N_2_	1408.5	765.195	1.1697	–
47	1-Hexanol D	C111273	C_6_H_14_O	1375.2	708.067	1.65737	Dimers
48	1-Hexanol P	C111273	C_6_H_14_O	1375.6	708.658	1.98876	–
49	(E)-2-Heptenal D	C18829555	C_7_H_12_O	1340.9	653.667	1.66735	Dimers
50	(E)-2-Heptenal M	C18829555	C_7_H_12_O	1342.1	655.441	1.25211	Monomers
51	1-Pentanol D	C71410	C_5_H_12_O	1262.8	526.538	1.53559	Dimers
52	1-Pentanol M	C71410	C_5_H_12_O	1263.4	527.625	1.25637	Monomers
53	2-Pentyl furan	C3777693	C_9_H_14_O	1238.6	485.078	1.25023	–
54	3-Octanone M	C106683	C_8_H_16_O	1228.9	469.345	1.30575	Monomers
55	3-Octanone D	C106683	C_8_H_16_O	1229.9	470.82	1.70414	Dimers
56	3-Methyl-but-3-en-1-ol	C763326	C_5_H_10_O	1199.8	425.097	1.4331	–
57	Heptaldehyde	C111717	C_7_H_14_O	1192.9	415.264	1.68781	–
58	Isopentyl alcohol D	C123513	C_5_H_12_O	1218.2	452.629	1.50494	Dimers
59	Isopentyl alcohol M	C123513	C_5_H_12_O	1,216	449.188	1.24207	Monomers
60	2,6-Dimethylpyrazine D	C108509	C_6_H_8_N_2_	1345.3	660.334	1.52233	Dimers
61	2,6-Dimethylpyrazine M	C108509	C_6_H_8_N_2_	1345.3	660.334	1.14674	Monomers
62	Hexan-2-one M	C591786	C_6_H_12_O	1138.5	344.359	1.19866	Monomers
63	1-Hydroxy-2-propanone	C116096	C_3_H_6_O_2_	1322.6	626.444	1.23317	–
64	2,5-Dimethylpyrazine	C123320	C_6_H_8_N_2_	1321.3	624.475	1.10184	–
65	Cyclohexanone	C108941	C_6_H_10_O	1303.5	599.035	1.16139	–
66	Ethyl hexanoate	C123660	C_8_H_16_O_2_	1245.7	496.842	1.33927	–
67	Hexan-2-one D	C591786	C_6_H_12_O	1141.3	347.658	1.50162	Dimers

RI, retention index; Rt, retention time; Dt, drift time.

According to Table 1, it can be seen that the content of the same VOCs differed significantly in different varieties of Fritillaria samples. Combined with the analysis of Table 1 and Figure 4, it can be concluded that a total of 67 VOCs were detected in six varieties of Fritillaria samples, including acids, ketones, esters, alcohols, aldehydes, furans, and pyrazine. This includes the monomers and dimers of some of the substances, in which the monomers and dimers identified had the same chemical formulas and CAS numbers, and only differed in their morphology.

### 3.3 Fingerprint spectrum analysis of Fritillaria samples from six different species

**Figure 5:**
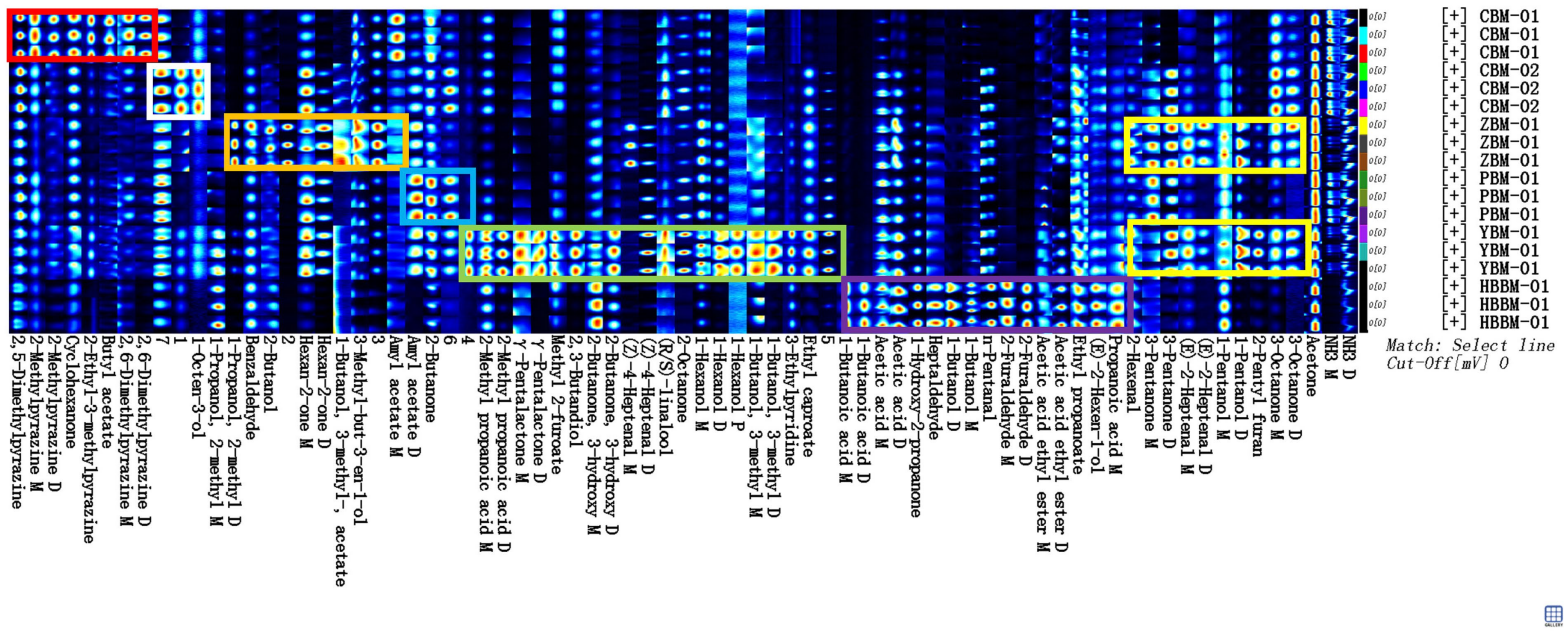
Gallery plot of different varieties of Fritillaria via GC-IMS.

Figure 5 shows the fingerprints of the VOCs in six samples of Fritillaria. Each row in the figure represents all the signal peaks selected in one sample, and each column represents the signal peaks of the same VOCs in different samples.

Comparative analysis of VOCs in samples CBM-01, CBM-02, ZBM-01, PBM-01, YBM-01, and HBBM-01 showed that, as shown in the red box, 2,5-dimethylpyrazine, 2-methylpyrazine, cyclohexane, 2-ethyl-3-methylpyrazine, butyl acetate, and 2,6-dimethylpyrazine were present in sample CBM-01 in higher levels. As shown in the white box, unknown peak 1, unknown peak 7, and 1-octen-3-ol were higher in the CBM-2 sample. As shown in the orange box, 2-methylpropanol, 2-butanol, unknown peak 2, hexan-2-one, isoamyl acetate, 3-methyl-but-3-en-1-ol, unknown peak 3, and benzaldehyde were higher in ZBM-1. As shown in the blue box, amyl acetate, 2-butanone, and unknown peak 6 were higher in the PBM-01 sample. As shown in the green box, unknown peak 4, 3-ethylpyridine, 2-methylpropionic acid, γ-pentalactone, 2-butanone,3-hydroxy, 2,3-butanedione, methyl 2-furoate, (Z)-4-heptenal, linalool, 1-octen-3-ol, hexanol, 2-octanone, ethyl caproate, 3-methylbutanol, and unknown peak 5 were higher in the YBM-01 sample. As shown in the purple box, butyric acid, propionic acid, furfural, acetic acid, 1-hydroxy-2-propanone, heptanal, butanol, glutaraldehyde, ethyl acetate, ethyl propionate, and (E)-2-hexenol were higher in the HBBM-01 sample. As shown in the yellow box, 2-hexenal, 3-pentanone, (E)-2-heptenal, pentanol, 2-pentylfuran, and 3-octanone were higher in both the ZBM-01 and YBM-01 samples.

This shows that GC-IMS can form a unique fingerprint, visualize the differences of VOCs in different varieties of Fritillaria, and quickly distinguish and identify different varieties of Fritillaria.

### 3.4 Chemometric analysis

Chemometrics is an emerging branch of chemistry formed by the intersection of chemistry and mathematics, computer science, etc. It is of great significance in the quality control and research and evaluation of traditional Chinese medicine by establishing a link between the measured values of a chemical system and the state of the system through statistical or mathematical methods (29). The chemometric analysis of VOCs in different varieties of Fritillaria is helpful for finding the differential components to identify their varieties.

#### 3.4.1 Principal component analysis

**Figure 6:**
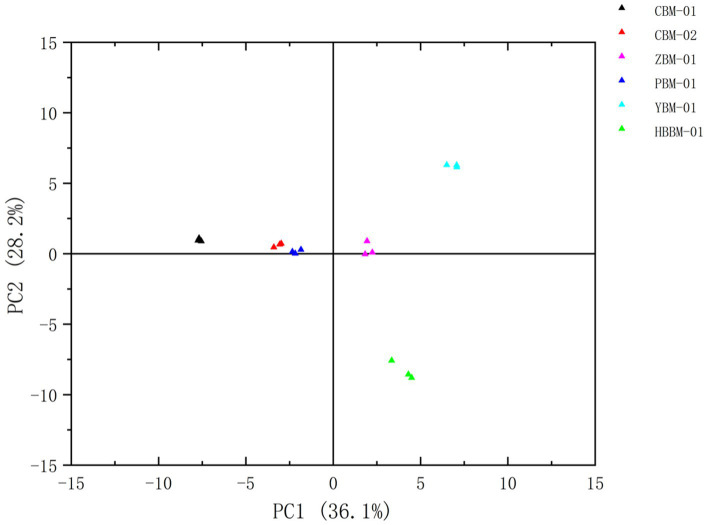
Plot of the PCA scores of VOCs in six species of Fritillaria.

Principal component analysis (PCA) is a commonly used unsupervised analysis method which reduces the dimensionality of the data while retaining as much original information as possible and can respond to the overall situation of the chemical measurement data for complex traditional Chinese medicine systems (30). As shown in the Figure 6, the PCA of the VOCs in the six varieties of Fritillaria was performed, and the results are shown in Figure 6, which shows that the six groups of samples were well separated. When the samples are close to each other, it indicates that the difference in VOCs between the samples is relatively small, and on the contrary, it indicates that there is a significant difference in the VOCs between them. The distance between CBM-01 and the other Fritillaria was relatively large, which indicates that PCA can distinguish Fritillariae cirrhosae bulbus from the other varieties of Fritillaria very well. The distance between CBM-02 and PBM-01 was closer, which indicates that the difference in the VOCs between the two kinds of samples is small, similar to a previous study. These results indicated significant differences in VOCs among the six varieties and showed that the PCA could effectively distinguish the four other species of Fritillaria from Fritillariae cirrhosae bulbus.

#### 3.4.2 Cluster analysis

**Figure 7:**
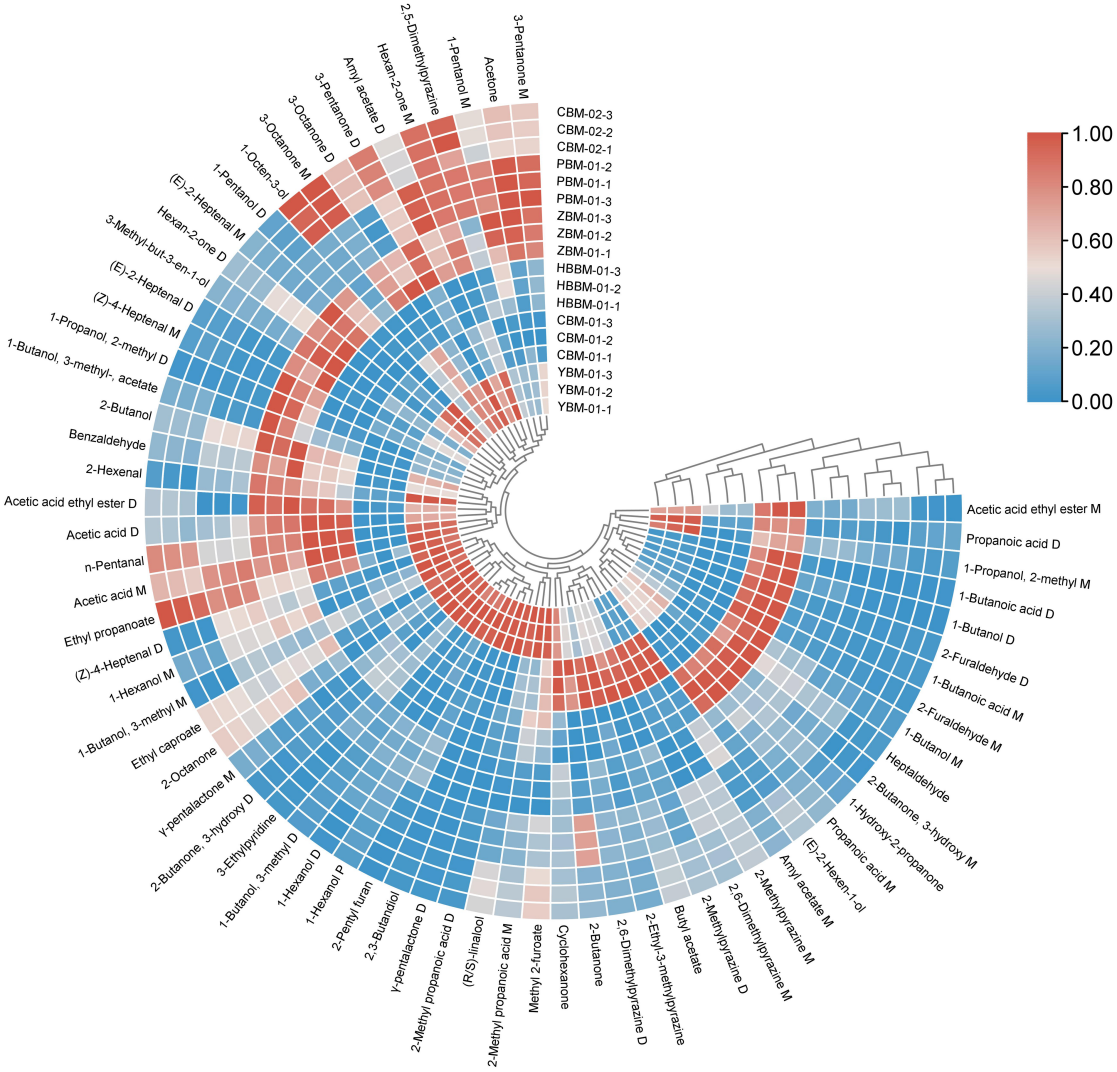
Cluster heat map of VOCs in six species of Fritillaria.

Heat maps can be used to reflect data via color changes, which can visually represent the differences between data using color shades (31). As shown in the Figure 7, the 67 VOCs identified with GC-IMS were clustered and analyzed, and the differences in the VOCs of the six varieties of Fritillaria were clearer and more explicit, as shown in Figure 7. The clustering heat map shows that there are obvious differences in the VOCs in different varieties of Fritillaria (32). Among them, the contents of acetic acid and ethyl propanoate substances in CBM-01 were significantly less than those in other Fritillaria. The contents of (Z)-4-heptenal, 1-propanol, 2-methyl, 3-methyl-but-3-en-1-ol, and hexan-2-one substances were higher in ZBM-01 than in the other Fritillaria, while the contents of 1-hexanol, 2,3-butandiol, and 2-methyl propanoic acid substances were higher in YBM-01 than in the other Fritillaria. The contents of, 1-butanol, 2-furaldehyde, and 1-butanoic acid substances were higher in HBBM-01 than in the other Fritillaria.

#### 3.4.3 Partial least-squares discriminant analysis

**Figure 8:**
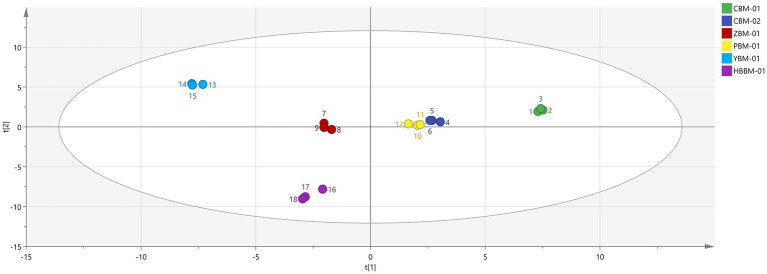
PLS-DA analysis of VOCs in 6 species of Fritillaria.

Partial least-squares discriminant analysis (PLS-DA) is a supervised pattern statistical analysis method that enables the visualization of complex data and is often used to deal with classification and discrimination problems (33). As shown in the Figure 8, to further explore the volatiles in different varieties of Fritillaria, PLS-DA modeling was performed with 69 VOCs identified as dependent variables and different varieties as independent variables. The results are shown in Figure 8. In this model, the six groups of samples were well separated from each other. The long distance between CBM-01 and other samples indicated that the VOCs of CBM-01 and other samples were significantly different. Meanwhile, a close distance between CBM-02 and PBM-01 samples can be observed, which is consistent with the results of PCA analysis.

**Figure 9:**
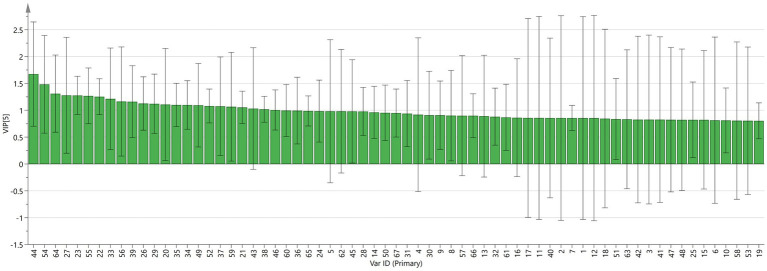
VIP values of the characteristic variables.

The variable importance projection (VIP) indicates the strength of influence and explanatory ability of the differential components on the classification discrimination of various samples, which is an important index for screening differential compounds. The larger the VIP value of the sample, the more significant the variable is in distinguishing the sample. As shown in Figure 9, 1-Octen-3-ol, 3-Octanone, and 2,5-Dimethylpyrazine are the most important components that affect the difference of six varieties of Fritillaria (Figure 9).

## 4 Discussion

In this study, a total of 76 substances, including 67 VOCs were detected. Firstly, based on the GC-IMS odor fingerprint data, it was preliminarily found that the types of VOCs in different varieties of Fritillaria are similar, but there are significant differences in content. So, we further utilize chemometrics to analyze all the obtained data. The results of PCA and PLS-DA indicate that the distribution of VOCs in samples of different varieties is relatively independent, which also confirms Fritillariae ussuriensis bulbus is often used as a substitute for Fritillariae cirrhosae bulbus because of its high similarity to Fritillariae cirrhosae bulbus. Although its clinical effect is not as good as that of Fritillariae cirrhosae bulbus, it is of great significance to alleviate the shortage of resources of Fritillariae cirrhosae bulbus. Through the results of the heat map, it can be seen that there are components with higher content in different varieties of Fritillaria, such as CBM-01 contains a lot of pyrazine compounds, 1-Octan-3-ol having the highest content in CBM-02, and 3-Methyl-but-3-en-1-ol, 1-Butanol-3-Methyl acetate and other substances having the highest content in ZBM-01. The results of the heat map also mutually confirm the conclusions of the odor fingerprint spectrum. That’s also why Fritillariae thunbergh bulbus has better efficacy in relieving coughs and resolving phlegm than Fritillariae cirrhosae bulbus, Fritillariae pallidiflorae bulbus, and Fritillariae ussuriensis bulbus, which is related to the fact that it contains more bemacropodin A and bemacropodin B (34).

Compared with the traditional methods such as DNA barcoding technique and high-performance liquid chromatography (HPLC), the determination of VOCs in different Fritillaria samples via GC-IMS to differentiate between species has greatly improved the identification method and efficiency, and the pretreatment of samples is more efficient and convenient. It is very helpful for quickly identifying different varieties of Fritillaria.

## 5 Conclusion

In this study, the VOCs of six species of Fritillaria were analyzed and systematically compared using GC-IMS. A total of 79 peaks were detected, with 67 VOCs identified, mainly including acids, ketones, esters, alcohols, aldehydes, furans, and pyrazine. Among them, CBM-01 contains a lot of pyrazine compounds, with 1-Octan-3-ol having the highest content in CBM-02, and 3-Methyl-but-3-en-1-ol, 1-Butanol-3-Methyl acetate and other substances having the highest content in ZBM-01.

Based on statistical analysis with chemometrics, it was found that there were some differences in the ion migration spectra of different Fritillaria varieties. Fingerprints were established based on the characteristic components fitted by the graph plug-in software, and after principal component analysis (PCA), cluster analysis (CA), and partial least-squares discriminant analysis (PLS-DA), we could effectively and quickly distinguish Fritillariae cirrhosae bulbus and its common other Fritillaria species. This provides a new method for the quality control and identification of quality relationships of Fritillariae cirrhosae bulbus. This study demonstrates that GC-IMS provides an important reference value for the identification and authenticity assessment of different varieties of Fritillaria, which is conducive to ensuring the quality of Fritillaria in the market, thus guaranteeing the safety and effectiveness of medication.

## Data availability statement

The original contributions presented in the study are included in the article/supplementary material, further inquiries can be directed to the corresponding authors.

## Author contributions

YD: Conceptualization, Data curation, Formal analysis, Investigation, Methodology, Software, Supervision, Writing – original draft. ShaL: Conceptualization, Data curation, Formal analysis, Investigation, Methodology, Software, Supervision, Writing – original draft. LY: Conceptualization, Data curation, Formal analysis, Funding acquisition, Writing – original draft. YH: Conceptualization, Data curation, Writing – original draft. XG: Formal analysis, Resources, Validation, Writing – original draft. YM: Methodology, Supervision, Validation, Writing – original draft. ShuL: Funding acquisition, Project administration, Writing – original draft, Writing – review & editing. DH: Formal analysis, Funding acquisition, Resources, Software, Supervision, Writing – original draft, Writing – review & editing.
